# Impact of 4 *Lactobacillus plantarum* capsular polysaccharide clusters on surface glycan composition and host cell signaling

**DOI:** 10.1186/1475-2859-11-149

**Published:** 2012-11-21

**Authors:** Daniela M Remus, Richard van Kranenburg, Iris I van Swam, Nico Taverne, Roger S Bongers, Michiel Wels, Jerry M Wells, Peter A Bron, Michiel Kleerebezem

**Affiliations:** 1 TI Food & Nutrition, Nieuwe Kanaal 9A, 6709 PA Wageningen,, The Netherlands; 2 NIZO food research, Kernhemseweg, 2, 6718 ZB Ede, The Netherlands; 3 Laboratory for Microbiology, Wageningen University, Dreijenplein 10, 6703 HB Wageningen, The Netherlands; 4 Purac, P.O. Box 20, , 4200 AA Gorinchem, The Netherlands; 5 Host-Microbe Interactomics Group, Wageningen University, De Elst 1, 6708 WD Wageningen, The Netherlands; 6 Kluyver Centre for Genomics of Industrial Fermentation, P.O. Box 5057, , 2600 GA Delft, The Netherlands

**Keywords:** Lactobacillus plantarum, Probiotic, Surface polysaccharides, Host cell signaling, TLR2 activation

## Abstract

**Background:**

Bacterial cell surface-associated polysaccharides are involved in the interactions of bacteria with their environment and play an important role in the communication between pathogenic bacteria and their host organisms. Cell surface polysaccharides of probiotic species are far less well described. Therefore, improved knowledge on these molecules is potentially of great importance to understand the strain-specific and proposed beneficial modes of probiotic action.

**Results:**

The *Lactobacillus plantarum* WCFS1 genome encodes 4 clusters of genes that are associated with surface polysaccharide production. Two of these clusters appear to encode all functions required for capsular polysaccharide formation (*cps2A-J* and *cps4A-J*), while the remaining clusters are predicted to lack genes encoding chain-length control functions and a priming glycosyl-transferase (*cps1A-I* and *cps3A-J*). We constructed *L. plantarum* WCFS1 gene deletion mutants that lack individual (Δ*cps1A-I*, *Δcps2A-J*, Δ*cps3A-J* and Δ*cps4A-J*) or combinations of *cps* clusters (Δ*cps1A-3J* and Δ*cps1A-3I*, Δ*cps4A-J*) and assessed the genome wide impact of these mutations by transcriptome analysis. The *cps* cluster deletions influenced the expression of variable gene sets in the individual *cps* cluster mutants, but also considerable numbers of up- and down-regulated genes were shared between mutants in *cps* cluster 1 and 2, as well as between mutant in *cps* clusters 3 and 4. Additionally, the composition of overall cell surface polysaccharide fractions was altered in each mutant strain, implying that despite the apparent incompleteness of *cps1A-I* and *cps3A-J*, all clusters are active and functional in *L. plantarum*. The Δ*cps1A-I* strain produced surface polysaccharides in equal amounts as compared to the wild-type strain, while the polysaccharides were characterized by a reduced molar mass and the lack of rhamnose. The mutants that lacked functional copies of *cps2A-J*, *cps3A-J* or *cps4A-J* produced decreased levels of surface polysaccharides, whereas the molar mass and the composition of polysaccharides was not affected by these cluster mutations. In the quadruple mutant, the amount of surface polysaccharides was strongly reduced. The impact of the *cps* cluster mutations on toll-like receptor (TLR)-mediated human nuclear factor (NF)-κB activation in host cells was evaluated using a TLR2 reporter cell line. In comparison to a *L. plantarum* wild-type derivative, TLR2 activation remained unaffected by the Δ*cps1A-I* and Δ*cps3A-J* mutants but appeared slightly increased after stimulation with the Δ*cps2A-J* and Δ*cps4A-J* mutants, while the Δ*cps1A-3J* and Δ*cps1A-3J,* Δ*cps4A-J* mutants elicited the strongest responses and clearly displayed enhanced TLR2 signaling.

**Conclusions:**

Our study reveals that modulation of surface glycan characteristics in *L. plantarum* highlights the role of these molecules in shielding of cell envelope embedded host receptor ligands. Although the apparently complete *cps* clusters (*cps2A-J* and *cps4A-J*) contributed individually to this shielding, the removal of all *cps* clusters led to the strongest signaling enhancement. Our findings provide new insights into cell surface glycan biosynthesis in *L. plantarum*, which bears relevance in the context of host-cell signaling by probiotic bacteria.

## Background

The Gram-positive bacterial cell envelope is a multilayered structure, which is mainly composed of peptidoglycan with embedded teichoic acids, proteins, and polysaccharides and which is essential to maintain cellular integrity and shape [1]. The molecules of the cell envelope collectively decorate the bacterial surface in a strain- and species-specific manner and facilitate important bacterial processes such as stress- and environmental-adaptation, surface colonization, and adhesion [2].

Lactic acid bacteria (LAB) are Gram-positive bacteria that can be encountered in a wide range of environmental niches, including a range of dairy and other food-raw material fermentations as well as the gastrointestinal (GI) tract of humans and animals. LAB have a clear industrial relevance, based on their role in food fermentation whereby they contribute to the preservation of raw-materials in foods, but also to the product’s flavor and texture [3]. Additionally, specific LAB are marketed as health-promoting organisms or probiotics [4]. Most LAB are able to synthesize extracellular polysaccharides, many of which consist of heteropolysaccharides built up from regular repeating oligosaccharide units that commonly contain the monosaccharides galactose, glucose, and in several cases also encompass rhamnose, *N*-acetyl-glucosamine, *N*-acetyl-galactosamine, mannose, and non-carbohydrate substitutions [5-8]. The repeating units are synthesized in the cytoplasm and assembled on the lipid carrier undecaprenyl phosphate by sequential transfer of monosaccharides from nucleotide sugars by specific glycosyltransferases. Membrane-associated and -assembled repeating-unit oligosaccharides are thought to be translocated across the cytoplasmic membrane and polymerized to polysaccharides by a dedicated transport and polymerization machinery [9,10]. The overall capacity for this type of polysaccharide synthesis is encoded by clusters of genes that are readily detectable by their conserved structural composition (Wzy-dependent polymer gene cluster) and are commonly transcribed as a single transcript [11], although exceptions have been reported [12]. These clusters are found in many Gram-positive bacteria and are probably best documented for *Streptococcus pneumonia*[13,14]. The first genes in these clusters (*wzd*, *wze*, *wzh*) are involved in modulation of capsule synthesis and form a tyrosine phosphoregulatory circuit that controls polymer production [14]. These regulatory gene-cassettes are commonly followed by a variable number of genes encoding glycosyltransferases, which are involved in repeat unit synthesis on the cytoplasmic face of the cell membrane [14]. Once synthesized, the repeat unit is flipped across the membrane by a dedicated flippase (Wzx) and subsequently polymerized (Wzy) to form the extracellular polysaccharide [14].

EPS produced by LAB has received a lot of attention based on their role as thickening agents that are naturally produced during fermentation and influence the textural properties of fermented dairy and non-dairy products [9,15-18]. Besides the industrial relevance of polysaccharides produced by LAB in product characteristics, they may also play a role in the interaction between microbes and the host intestinal mucosa. While in pathogenic bacteria such as *Streptococcus pneumoniae*, polysaccharide capsules are extensively studied and were shown to play important roles in virulence by inhibiting opsonization and phagocytosis and are employed frequently as important serotyping antigens for epidemiological studies [19,20], the role of cell surface polysaccharides of commensal or probiotic bacteria is far less understood. In *Lactobacillus rhamnosus* GG, biosynthesis of the high-molecular-weight, galactose-rich surface polysaccharide molecules negatively affects the bacterial capacity to bind to intestinal epithelial cells, which may be due to the shielding of adhesins on the bacterial cell surface [21]. Additionally, surface polysaccharides may also contribute to protection against intestinal innate immune factors such as the antimicrobial peptide LL-37 [22]. A direct role in host signaling has been proposed for purified surface polysaccharides of *Lactobacillus casei* Shirota that were shown to mediate the suppression of pro- inflammatory responses in macrophages [23]. Moreover, several polysaccharide biosynthesis-related genes of *Lactobacillus plantarum* were up-regulated *in vivo* in the GI-tract of mice and humans [24], and a recent study tentatively correlated elevated expression of *cps* genes to enhanced survival under GI-tract mimicking conditions in this species (van Bokhorst-van de Veen, unpublished observations).

Here, we characterize the 4 *cps* gene clusters encoded by *L. plantarum* WCFS1, an extensively studied model organism for probiotic lactobacilli, aiming to identify bacterial molecules involved in microbe-host interactions with relevance for probiotic functions [25]. We constructed various *cps*-cluster deficient strains, including single (Δ*cps1A-I*, Δ*cps2A-J*, Δ*cps3A-J*, Δ*cps4A-J*), triple (Δ*cps1A-3J*), and quadruple (Δ*cps1A-3J*, Δ*cps4A-J*) deletion mutants. The consequences of *cps* cluster mutation were studied at the level of whole genome transcriptome profiles, and by determining the amount, molar mass and chemical composition of the surface polysaccharide fraction of the mutants in comparison to the wild-type strain. Finally, the impact of the *cps* cluster mutations on toll-like receptor (TLR)-dependent human nuclear factor (NF)-κB activation in host cells was evaluated using a TLR2 reporter cell line.

## Results and discussion

### CPS biosynthesis cluster organization in *L. plantarum* WCFS1

**Figure 1:**
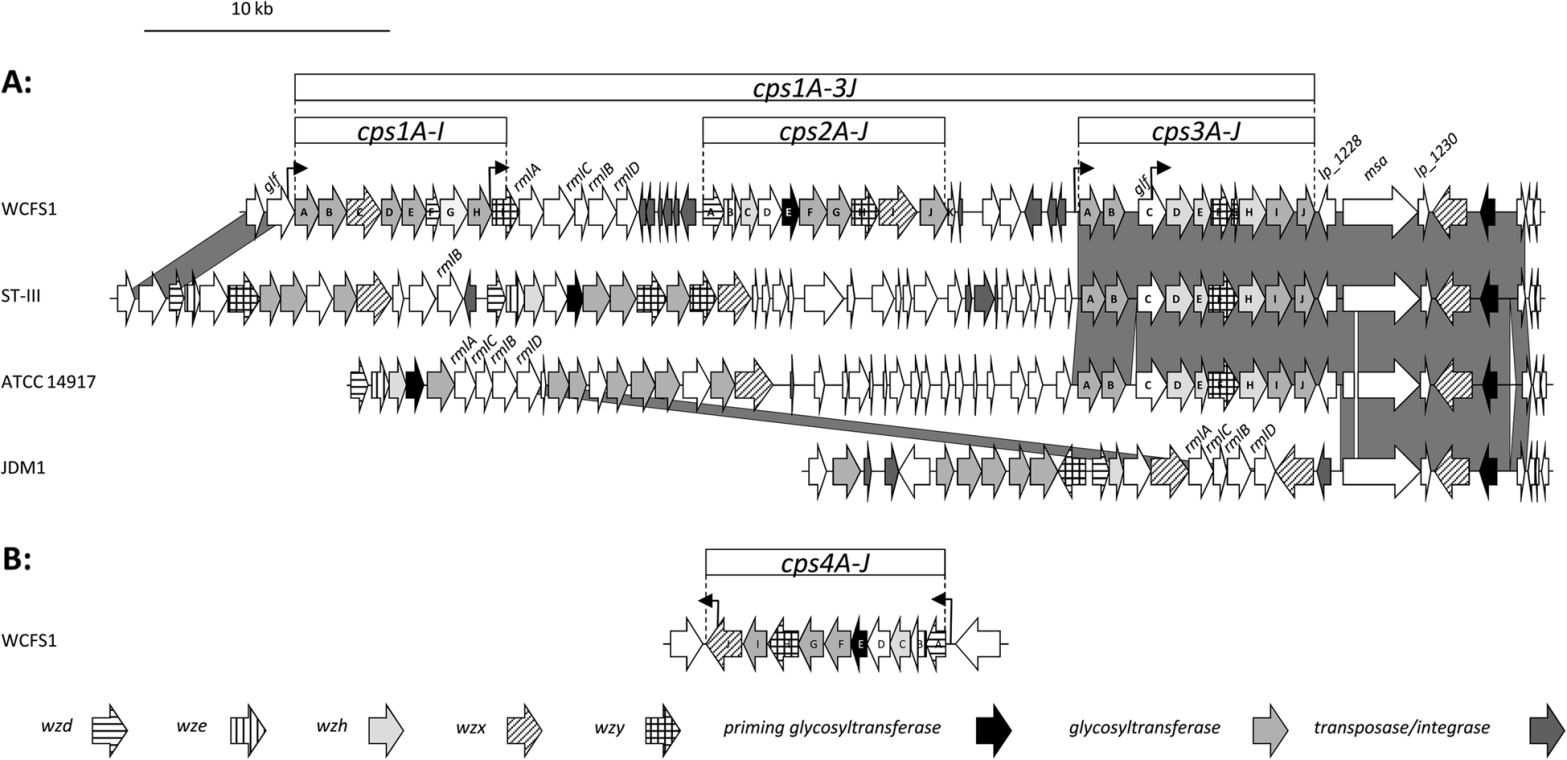
**Genetic organization of polysaccharide biosynthesis gene clusters in *L. plantarum *strains.** (**A**) Genetic organization of the *L. plantarum* WCFS1 polysaccharide biosynthesis gene clusters 1, 2, and 3 (AL935263; *lp_1176* through *lp_1234*) and comparison with the corresponding regions of *L. plantarum* strains ST-III (NC_014554; LPST_C0945 through LPST_C0997), JDM-1 (NC_012984; JDM1_1015 through JDM1_1041), and ATCC 14917 (ACGZ02000014; HMPREF0531_11685 through HMPREF0531_11729; ACGZ02000010; HMPREF0531_11316 through HMPREF0531_11319). Dark-grey colored connecting blocks indicate regions of high sequence conservation identified in the genomes of the indicated strains. The white area connecting *msa* homologues gene of ST-III and JDM-1, indicates that this gene is separated on two contigs in the draft genome sequence available for ATCC14917. (**B**) Genetic organization of the *L. plantarum* WCFS1 polysaccharide biosynthesis gene cluster 4. The corresponding regions are conserved in *L. plantarum* ST-III, ATCC 14917, and JDM1 sharing 99%, 99%, and 97% nucleotide identity with WCFS 1, respectively. Arrows indicate predicted promoters with e-value ≤ 10^-5^[27].

The *L. plantarum* WCFS1 genome contains two regions with surface-associated polysaccharide biosynthesis genes that are designated *cps* genes (Figure 1A and B). One region of 49 kb contains three gene clusters separated by transposase genes and has been identified as a genomic life-style island with high variability between *L. plantarum* strains [26]. Of these three gene clusters, *cps1* and *cps2* are identified only in strain WCFS1 and not in the other completely sequenced strains of the same species, while gene cluster *cps3* is conserved in ST-III and ATCC 14917 but not in JDM1 (Figure 1; Additional file 1). A second region of 14 kb comprises the WCFS1 *cps4* gene cluster and is conserved in other *L. plantarum* strains (Additional file 1). Clusters 2 and 4 have a typical structure of a Wzy-dependent polymer gene cluster (Figure 1; Additional file 1). The first three genes (*cps2ABC* and *cps4ABC*) are homologous to the typical components of the tyrosine kinase phospho-regulatory circuit involved in control of capsule synthesis [14]. The fourth gene in both gene clusters is predicted to encode an UDP-*N*-acetylglucosamine 4-epimerase catalyzing the interconversion between UDP-*N*-acetyl-d-glucosamine and UDP-*N*-acetyl-d-galactosamine (*cps2D*, and *cps4D*). The fifth gene is predicted to encode the priming glycosyltransferase catalyzing the transfer of a sugar-1-phosphate from a UDP-sugar to the undecaprenyl-phosphate, the first step in the synthesis of the repeat unit (*cps2E* and *cps4E*). The *cps* clusters 2 and 4 contain three additional genes with high sequence similarity to glycosyltransferase genes (*cps2FGJ* and *cps4FGI*), indicating that the encoded polysaccharides would be made up of quatro-saccharide repeat units. The 3’ regions of both clusters encode homologues of the typical flippase (*cps2I* and *cps4J*) and polymerase (*cps2H* and *cps4H*) functions required for capsule synthesis [14].

The organization of clusters 1 and 3 is different from that of clusters 2 and 4. Cluster 3 appears to lack clear homologues of the chain-length modulator genes *wzd*, *wze*, and *wzh*. However, it is tempting to speculate that *cps3D*, *cps3E*, and *cps3H* serve such function, as their gene products show low sequence similarities to several polysaccharide biosynthesis proteins of unknown functions and Cps3E shares distant homology (26% identity) with the N-terminus of *Clavibacter michiganensis* subsp. *michiganensis* NCPPB 382 Wzc tyrosine kinase (functional homologues *wzd* and *wze*; NCBI accession number YP_001221462). The *cps3 wzy* homologue is split in two by a frame-shift caused by a single nucleotide insertion immediately upstream of the *cps3F* stop codon. It is unclear if a functional Wzy protein can be composed of Cps3F and Cps3G. The cluster contains three predicted glycosyltransferase genes but no priming glycosyltransferase gene. The presence of an acetyltransferase gene indicates that acetylation of the repeat units might take place. A set of genes encoding two transcriptional regulators and a mannose-specific adhesin protein separates the *cps3* gene cluster from a polysaccharide polymerase-like (*lp_1231*) and priming glycosyltransferase (*lp_1233*) gene (Figure 1). These genes could complete the polysaccharide synthesis machinery of *cps3*, which would then be predicted to be involved in the synthesis of a polysaccharide made up of acetylated quatro-saccharide repeat units. Notably, in *Lactobacillus rhamnosus*, exopolysaccharide gene clusters the priming glycosyltransferase genes are also separated from the body of the polysaccharide gene cluster [12].

The *glf* gene of *cps* cluster 1 is predicted to encode an UDP-galactopyranose mutase catalyzing the interconversion of UDP-galactopyranose and UDP-galacto-1,4-furanose. The *glf* gene encodes a protein with 86% identity to the protein encoded by the cluster 3 *glf* gene. The *cps1* cluster contains 5 predicted glycosyltransferase genes but like the *cps3* cluster, it appears to lack a priming glycosyltransferase gene. In addition, it contains a predicted acetyltransferase gene, *wzx*, *wzy*, and *wzd* homologues but no *wze* and *wzh* homologues. Expression of the *cps1* cluster is predicted to produce a polysaccharide comprising an acetylated hexa-saccharide repeat unit.

A functional role of the *cps* 1 and 3 clusters in production of surface glycans in *L. plantarum* WCFS1 is supported by the impact of individual deletion of either the *cps1* or *cps3* genecluster on the surface glycan characteristics (see below). Notably, the lack of a predicted priming glycosyltransferase and in both the *cps3* and *cps1* clusters could imply that the glycosyltransferases encoded by these clusters interact with the undecaprenylphosphate-sugars derived from other pathways (e.g., *cps* clusters 2 and 4, or more remotely encoded priming glycosyltransferases like the *lp_1233*-encoded function). However, an additional or alternative role of the *cps* geneclusters 1 and 3 in glycosylation of other surface molecules like teichoic acids and/or proteins as has been hypothesized before [2] can’t be excluded at this stage.

### Deletion of *cps* clusters impacts on the *L. plantarum* transcriptome

**Figure 2:**
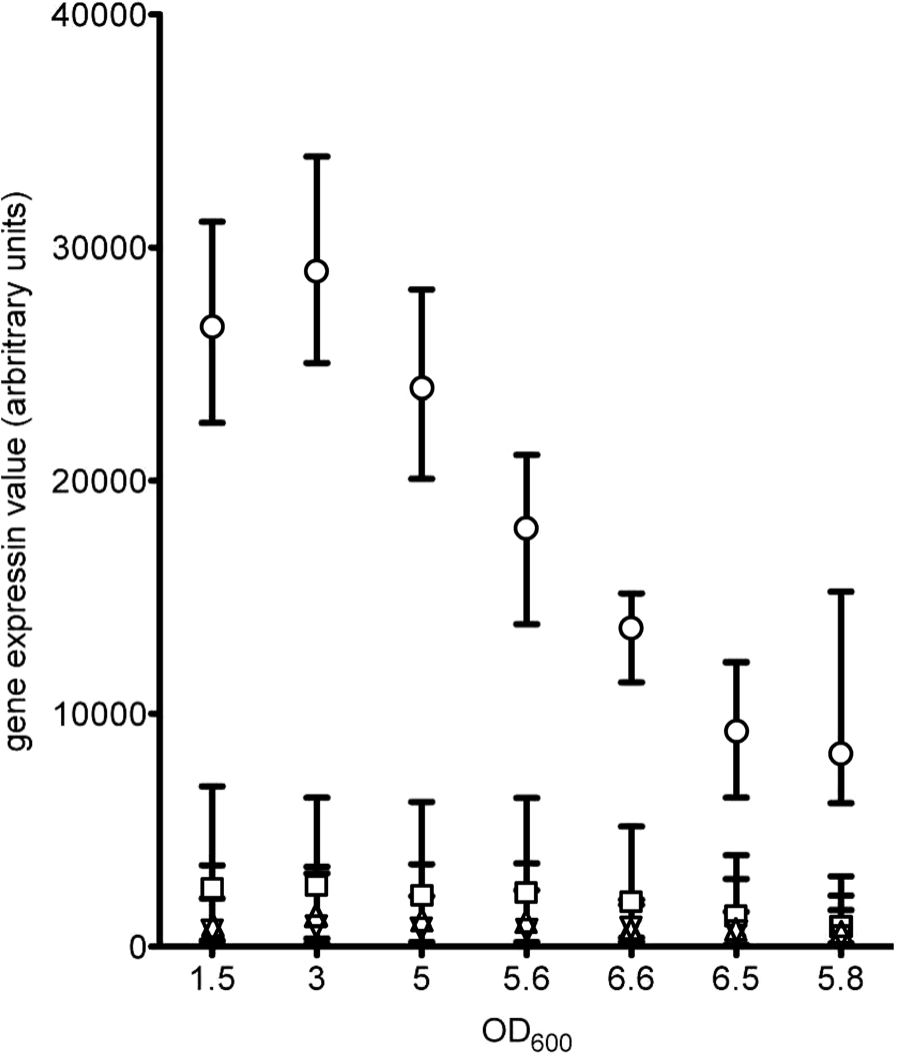
**Growth phase dependent gene expression levels of individual *cps *gene clusters in *L. plantarum *WCFS1.** Data is represented as average expression of *cps* genes of cluster 1 (circles), 2 (squares), 3 (upward pointing arrowheads) and 4(downward pointing arrowheads). Error bars represent the range of expression between the highest and lowest expressed gene in the different clusters.

We previously generated global growth phase-dependent gene expression profiles of *L. plantarum* WCFS1 ranging from mid-logarithmic to late stationary growth phase (Daniela M. Remus, Fabrizia Fusetti, Jurgen Karczewski, Roger S. Bongers, Irene Konings, Bert Poolman, Maria L. Marco, Paul de Vos, Jerry M. Wells, Michiel Wels, Peter A. Bron and Michiel Kleerebezem, unpublished observations). Analysis of the *cps* cluster-related gene expression patterns revealed that the *cps1A-I* cluster-associated genes were highly expressed under laboratory conditions, whereas the genes of *cps* clusters *2A-K*, *3A-I* and *4A-J* were expressed at much lower levels (Figure 2).

Genome-wide transcriptional profiles of the single *cps* cluster deletion strains were generated, confirming the complete abolishment of expression of the genes affected by the mutation, and thus establishing the integrity of the mutants. These transcriptome analyses also revealed that *cps* cluster deletion affected the expression of variable gene sets in the individual *cps* cluster mutants as compared to the wild-type strain. Deletion of *cps* cluster 1 or 2 affected the largest group of genes (Table 1), whereas the transcriptome impact of deletion of cluster 4 was considerably smaller, and deletion of cluster 3 appeared the smallest (Table 1). Intriguingly, the transcriptome changes elicited upon deletion of *cps* clusters 1 or 2 as compared to the wild-type strain, shared a substantial number of downregulated genes (87 shared repressed genes) for which the encoded functions are associated with transport and metabolism of certain amino acids, including serine, glutamate, aspartate, asparagines, methionine, cysteine, and the ABC-transporter for the branch chain amino acids (Additional file 2). Notably, genes that were consistently upregulated upon deletion of cps clusters 1 and 2 (30 upregulated genes shared, Additional file 2) included some that encoded amino acid related functions, including a di- and tri-peptide transport system as well as specific glutamine and a facilitator-family branched-chain amino acid transport systems, but also several genes with predicted cell-envelope associated functions like several extracellular proteins, and genes involved in amino-sugar synthesis and teichoic acid decoration. Remarkably, of the 20 upregulated genes observed in the *cps* cluster 3 deletion mutant relative to the wild-type strain, 11 were shared with the upregulated gene set found in the *cps* cluster 4 deletion mutant (Additional file 2). These genes included those encoding metabolic functions associated with pyruvate discipation or its control like, L-lactate dehydrogenase (*ldhL2*), pyruvate dehydrogenase (*pdhA*, *pdhB*; also activated in the *cps* 2 deletion strain), NADH oxidase (*nox5*), but also several carbohydrate degradation and transport functions like an α-amylase (*malS*), and the genetically linked and correlated transport functions for maltodextrins (*mdxG*), as well as two 6-phospho-beta-glucosidases (*pbg5*, and *pbg4*). Among these 6-phospho-beta-glucosidase encoding genes, the *pgb4* gene appeared to be induced in all *cps* cluster deletion strains, a feature shared by only two additional genes that appeared to be consistently downregulated in all mutants, and encode a prephenate dehydrogenase (*tyrA*), and a sodium-coupled N-acetylneuramidate transporter (*lp*_*3563*). In addition, expression of a FAD/FMN-containing dehydrogenase encoding gene (*lp_0291*) appeared to be differentially affected in the 4 *cps* deletion strains, i.e., downregulated upon deletion of *cps* cluster 1 or 2, but upregulated when *cps* cluster 3 or 4 is deleted. Importantly, compensatory activation of one of the alternative *cps* clusters was not observed in any of the *cps* cluster deletion mutants. These results show that especially deletion of *cps* cluster 1 or 2 elicited pleiotropic transcriptome consequences, which appeared to be centered on genes with functions associated with metabolism and transport of various amino acids, but also included genes encoding several other functions. The observation that deletion of the capacity to produce CPS affected the expression of genes encoding specific transport and metabolism functions, may suggest that the presence of polysaccharides in the cell envelope plays a role in the access that the bacteria have to nutrients from the environment. Polysaccharides may function as macromolecules that sequester nutrients and thereby facilitate their import [28,29], or alternatively they may form a capsular structure that surrounds the cell and retards or inhibits diffusion or transport of nutrients towards the membrane surface and thereby reduces trans-membrane transport efficiencies.

**Table 1:** **Number of genes, which were significantly changed in response to individual *cps *cluster deletions (false discovery rate adjusted p-value ≤ 0.05)**

	***L. plantarum cps *mutant strains**			
	NZ3548cm Δ*cps1A-I*	NZ5333ACm Δ*cps2A-J*	NZ3549Cm Δ*cps3A-J*	NZ3534Cm Δ*cps4A-J*
Upregulated	62	84	20	58
Downregulated	124	158	20	49
Total	186	242	40	107

### Impact of individual *cps* cluster mutations on *L. plantarum* surface glycan characteristics

Surface polysaccharides of *L. plantarum* WCFS1 and the *cps* cluster deletion mutants were isolated to assess their monosaccharide composition (Table 2). The results clearly demonstrated that the wild-type strain produces significant amounts of surface polysaccharides. Deletion of cluster 1 did not impact on the total amount of surface polysaccharides produced, which is remarkable in the light of the relatively high expression of the *cps1A-I* gene cluster in the wild-type (Figure 2) and the lack of compensatory expression changes of clusters 2, 3, or 4 in the *cps1A-I* mutant. However, the deletion of cluster 1 led to a decreased molar mass of the isolated polysaccharides and influenced the monosaccharide composition, i.e. led to a reduced relative amount of galactose and to a complete lack of rhamnose. This observation establishes that the *cps1* cluster is functional and leads to production of a specific polysaccharide in *L. plantarum* WCFS1. In addition, the lack of rhamnose in the surface polysaccharide fraction obtained for strain NZ3548Cm *(*Δ*cps1A-I*) is in agreement with the annotation of *cps1H* as the only rhamnosyltransferase found in all *cps* clusters. As the expression of the *rfb* genes (genetically linked to the *cps1* cluster) was not abolished in response to *cps1A-I* deletion (even slightly increased expression), it is likely that indeed the deletion of *cps1H* has led to the loss of rhamnose in the surface polysaccharide fraction.

**Table 2:** **Surface glycan composition of *L. plantarum *WCFS1 and its *cps *cluster deletion mutant derivatives**

** Sugar (% of total sugars)**	***L. plantarum *strains**					
	WCSF1	NZ3548Cm Δ*cps1A-I*	NZ5333ACm Δ*cps2A-J*	NZ3549Cm Δ*cps3A-J*	NZ3534Cm Δ*cps4A-J*	NZ3680Cm Δ*cps1A-3J*, Δ*cps4A-J*
Rhamnose	5.40	n.d.*	3.08	3.81	4.29	n.d.*
Glucosamine	3.28	5.04	3.28	3.45	2.95	3.93
Galactose	17.39	0.86	8.96	15.46	14.82	1.09
Glucose	27.98	23.81	29.16	25.28	27.03	22.22
Galacturonic-acid	45.66	69.79	55.52	52.00	50.92	72.23
Molar mass (kg/mol)	28.93	16.5	29.36	27.79	26.82	17.64
Polysaccharides isolated (mg/L)	31.52	30.62	11.98	15.68	12.88	3.82

*n.d. stands for ‘not-detected’.

Despite the low expression levels of clusters 2, 3 and 4 (Figure 2), deletion of each cluster reduced the production of surface polysaccharides, indicating their respective contributions to the overall surface polysaccharides produced by the wild-type strain (Table 2). The polysaccharides isolated from these mutants had a similar molecular mass as the polysaccharides isolated from the wild-type strain, indicating a particular role of the *cps* cluster 1 in polysaccharide chain length determination. Deletion of cluster 2 reduced the relative abundance of galactose in the surface polysaccharides, indicating the presence of this sugar in the *cps2*-encoded polysaccharide. In contrast, deletion of clusters 3 or 4 did not significantly affect the monosaccharide compositions of the polysaccharides produced. Notably, all polysaccharide molecules detected in the *cps2A-J*, *cps3A-J* and *cps4A-J* mutant strains contained rhamnose, whereby the presence of this monosaccharide in the surface polysaccharide fraction appears to be exclusively dependent on the *cps1A-I* cluster.

Deletion of all 4 *cps* clusters (Δ*cps1A-3J*, Δ*cps4A-J*) led to a substantial reduction (approximately 90% reduction in comparison to the wild-type) of the overall amount of surface polysaccharides isolated, which completely lacked rhamnose and showed a reduced relative amount of galactose, analogous to the Δ*cps1A-I* strain. As this mutant is expected to lack the genetic capacity to produce these typical *wzy*-dependent polysaccharides, the surface glycans isolated from this strain may derive from other surface polymers that are synthesized via other mechanisms. These remaining polysaccharides contain glucose, glucosamine, and galacturonic acid and might derive from teichoic acids, glycosylated proteins, and/or peptidoglycan. Remarkably, mutation of the individual *cps* clusters did not lead to an apparent phenotypic change in terms of growth or cell morphology, while the deletion of all four clusters (Δ*cps1A-3J*, Δ*cps4A-J*) caused aggregation of cells and rapid sedimentation (data not shown), which may be explained by increased cell surface-hydrophobicity due to reduced amounts of the typically hydrophilic surface polysaccharides [30].

### Deletion of *cps* clusters influences *L. plantarum* TLR2-mediated NF-κB activation

**Figure 3:**
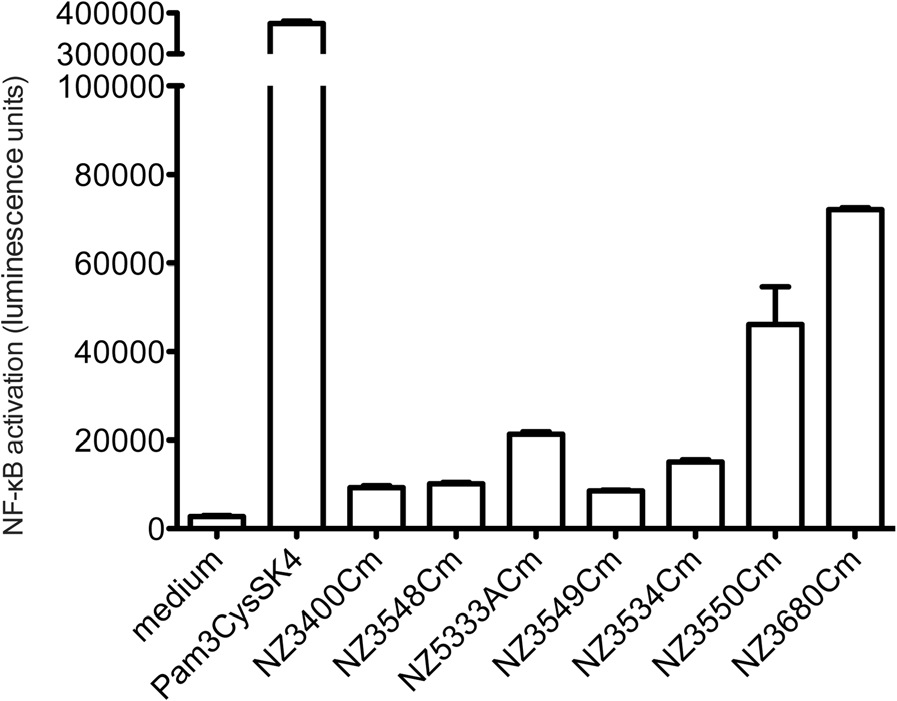
**Impact of *cps *cluster deletion on host cell signaling measured by a luminescence reporter in TLR2-expressing HEK-293 cells.** Data is represented as average +/− the range between 2 independently grown bacterial cultures (15 CFU/HEK-293 cell) of the *L. plantarum* wild-type like derivative (NZ3400Cm) and *cps* deletion mutants (NZ3548cm/Δ*cps1A-I*, NZ5333ACm/Δ*cps2A-J*, NZ3549Cm/Δ*cps3A-J*, NZ3534Cm/*Δcps4A-J,* NZ3550Cm/ Δ*cps1A-3J* and NZ3680Cm/Δ*cps1A-3J*, Δ*cps4A-J*).

The effects of *cps* cluster deletion on host cell signaling were examined in HEK-293 reporter cell lines stably expressing human Toll-like receptor (TLR)2, which harbor a reporter plasmid containing firefly luciferase under the control of the human nuclear factor (NF)-κB promoter. HEK-293 cells do not normally produce TLRs and have been previously shown to be unresponsive to several microbial-associated molecular patterns (MAMPs) [31,32] HEK-293 cells transiently transfected with only the NF-κB reporter plasmid (pNiFTY) were unresponsive to TLR2 agonists of *L. plantarum* demonstrating the requirement of human TLR2 in this signaling pathway (data not shown). An *L plantarum* wild-type like derivative (NZ3400Cm) activated TLR2-dependent NF-κB (Figure 3). Overall, activation of NF-κB remained unaffected by Δ*cps1A-I* and Δ*cps3A-J* but was slightly increased after stimulation by the Δ*cps2A-J* and Δ*cps4A-J* mutants, which are the strains that displayed the largest reduction in the amount of surface polysaccharides produced. Notably, exposure of TLR2-expressing HEK-293 cells to Δ*cps1A-3J* and Δ*cps1A-3J,* Δ*cps4A-J* elicited the strongest responses and clearly activated NF-κB. Although *cps4A-J* appears to be expressed at very low levels, its deletion in the Δ*cps1A-3J* background led to a substantial increase of TLR2-mediated NF-κB activity of the resulting strain, demonstrating that deletion of all *cps* clusters and the concomitant severe reduction of surface polysaccharide production leads to enhanced release and/or exposure of TLR2-agonists. These results suggest a role of surface polysaccharides in the shielding of other *L. plantarum* cell envelope MAMPs such as lipoproteins and teichoic acids that could activate TLR signaling, as was previously proposed for *Lactobacillus rhamnosus* GG [21].

## Conclusions

In *L. plantarum,* surface polysaccharide biosynthesis is encoded by 4 gene clusters, which independently contribute to the overall surface polysaccharides produced in this bacterium. Transcriptome analysis revealed that *cps* cluster deletion affected different sets of genes in the individual *cps* cluster mutants, whereas substantial numbers of regulated genes were shared between *cps* cluster mutant 1 and 2. Surface polysaccharide analysis revealed that the individual clusters influenced specific features of the polysaccharides produced, i.e. the amount, composition and molar mass. Although some of the individual mutants moderately affected TLR2-mediated NF-κB signaling, deletion of all clusters elicited a drastically increased NF-κB activation. In conclusion, the *cps* cluster encoded surface polysaccharides contribute to the *L. plantarum* cell surface architecture, and probably reduce release and/or exposure of TLR2-activating bacterial molecules.

### Bacterial culture conditions

*Escherichia coli* strain TOP-10 (Invitrogen, Carlsbad, USA), which was used as intermediate cloning host, was grown aerobically in TY medium. *L. plantarum* WCFS1 [33,34] and its derivatives (Additional file 3) were either cultivated in Mann-Rogosa Sharpe (MRS; Merck, Darmstadt, Germany) or in 2-fold concentrated (2 ×) chemical defined media (CDM) [35] supplemented with 1.5% (wt/vol) glucose without agitation. All bacteria were grown at 37°C and when appropriate, the media were supplemented with antibiotics; for *E*. *coli* and *L. plantarum* chloramphenicol was added at 10 μg/mL, and during replica plating of *L. plantarum* chloramphenicol and erythromycin were added at final concentrations of 10 μg/mL and 30 μg/mL, respectively.

### DNA manipulation techniques

Plasmid DNA was isolated from *E. coli* using Jetstar columns following the manufacturer’s instructions (Genomed GmbH, Bad Oberhausen, Germany). Restriction endonucleases (Fermentas, St. Leon-Rot, Germany), KOD-DNA polymerase (Toyobo, Osaka, Japan) and T4 DNA ligase (Invitrogen, Carlsbad CA, USA) were used as recommended by the manufacturers. Primers were obtained from Sigma Aldrich (Zwijndrecht, The Netherlands), and DNA sequencing reactions were performed at BaseClear (Leiden, The Netherlands). Chromosomal DNA isolation, preparation of electrocompetent cells and DNA transformation of *L. plantarum* were performed as described previously [36,37].

### Construction of *cps* cluster deletion mutants

Plasmids, primers and strains used in this study are listed in Additional file 3. The *cps* deletion mutants were constructed according to previously described methods [38], by which the target *cps* clusters were replaced by a chloramphenicol acetyltransferase (*cat*) gene cassette. In this study a derivative of the commonly used mutagenesis vector pNZ5319 [38], designated pNZ5319TAG (Bron *et al*., unpublished observations) was used that introduces a unique DNA-tag into the chromosome during gene deletion, which can be used for detection purposes (Bron *et al*., unpublished observations). The 5’- and 3’- flanking regions of the individual *cps* gene clusters (*cps1A-I*, *cps2A-J*, *cps3A-J, and* Δ*cps4A-J*) and of the *cps1A-3J-* and the H-locus- spanning regions were amplified by PCR. The amplicons representing the flanking regions of the target *cps* clusters and the H-locus were subsequently joined by a second PCR to the tag-lox66-F3/tag-lox71-R3 or tag-lox66-F2/tag-lox71-catR2 cassette, respectively. The resulting amplicons were cloned into SwaI-Ecl136II digested pNZ5319TAG. The obtained mutagenesis plasmids were integrated into the *L. plantarum* WCFS1 chromosome by double cross over replacement of the target gene (clusters) by the *cat* cassette, yielding the deletion mutant strains NZ3548Cm (Δ*cps1A-I*), NZ5333ACm (Δ*cps2A-J*), NZ3549Cm (Δ*cps3A-J*), and NZ3550Cm (Δ*cps1A-3J*), as well as the tagged strain NZ3400Cm (*lp_2681*-P_32_*cat-lp_2683*). For construction of the quadruple mutant (Δ*cps1A-3I*, Δ*cps4A-J*), the mutagenesis plasmid pNZ3550 (Additional file 3) was integrated into the Δ*cps4A-J* chromosome, in which the *cat* cassette was removed by the temporal expression of the Cre recombinase [38], yielding the deletion mutant strain NZ3534Cm (Δ*cps1A-3I*, Δ*cps4A-J*) [39]. The anticipated genotype of all mutants was confirmed by PCR using primers flanking the sites of recombination.

### RNA isolation

*L. plantarum* and its *cps*-cluster deficient derivatives were grown in 2 × CDM and RNA was isolated according to previous described methods [40,41]. In short, following methanol quenching [42], cells were harvested by centrifugation (6000 × g, 20 min, 4°C), resuspended in 400 μL ice-cold CDM medium and transferred to tubes containing 500 μL phenol/chloroform solution (4:1 [v/v]), 30 μL 10% sodium dodecyl sulfate, 30 μL 3 M sodium acetate (pH 5.2), and 0.5 g zirconium beads. Cells were disrupted by bead beating using a Savant FastPrep FP120 instrument (Qbiogen Inc., Illkirch, France), and RNA was purified from the aqueous phase using the High Pure Isolation Kit (Roche Diagnostics, Germany). RNA concentration and purity were determined using A260 and A280 measurements using a ND-1000 spectrometer (NanoDrop Technologies Inc., Wilmington, United States), and RNA quality was verified with a 2100 Bioanalyzer (Agilent Technologies, Amstelveen, the Netherlands). Samples that displayed a 23S/16S RNA ratio equal or superior to 1.6 were used for labeling.

### Transcriptome analysis and interpretation

3 μg RNA was used for cDNA synthesis. Cyanine-3 (Cy3) and cyanine-5 (Cy5) cDNA labeling was performed as described previously [43], using the CyScribe Post-Labeling and Purification kits according to the manufacturer’s instructions (Amsersham Biosciences, Buckinghamshire, UK). Cy-dye-labeled cDNAs (0.5 μg each) were hybridized to *L. plantarum* WCFS1 printed-oligonucleotide DNA microarrays (Agilent Technologies, Amstelveen, the Netherlands). The array design and transcriptome data were deposited under platform GPL13984 and accession number GSE34690 in NCBI’s Gene Expression Omnibus (GEO) [44,45] at http://www.ncbi.nlm.nih.gov/geo/. Hybridization and scanning procedures were performed as previously described [43]. Slide scanning was carried out at several photo multiplier tube (pmt) values, and the optimal scan of each individual microarray was selected on the basis of signal distribution (combination of a low number of saturated spots and a low number of low signal spots). The data were normalized using the Lowess normalization as available in MicroPrep [46]. For statistical significance, Benjamini and Hochberg’s False Discovery Rate (FDR) was used [47], with a FDR-adjusted p-value cutoff of 0.05, employed for genes showing at least 2-fold altered expression.

### Surface polysaccharide isolation and determination of glycan composition

Surface polysaccharides were isolated and characterized according to previously described methods [48,49]. *L. plantarum* WCFS1 and its mutant derivatives were grown in 2 × CDM until late stationary phase. After growth, cultures were incubated at 55°C for 1 h, followed by pelleting of the bacterial cells (6000 x g, 15 min, room temperature). The supernatants were supplemented with erythromycin (30 μg/mL), transferred to dialysis tubes (molecular weight cutoff of 12–14000 Da, Fisher Scientific, Landsmeer, The Netherland) and dialyzed overnight against running tap water followed by dialysis for 4 h against deionized water. The dialyzed samples were freeze-dried and stored at −20°C until further analysis.

Samples were dissolved in eluent (100 mM NaNO_3_ + 0.02% NaN_3_), and polysaccharides were separated by size exclusion chromatography (SEC), light scattering was measured at 632.8 nm, UV-adsorption of proteins was measured at 280 nm, viscosity was measured with a viscosity detector (ViscoStar, Wyatt Technologies, Santa Barbara, USA), and sample concentrations were measured by determining their refraction index. During SEC, polysaccharide peaks were collected and hydrolyzed with 2 M trifluoroacetic acid (TFA), dried and dissolved in water. The quantitative monosaccharide composition of the polysaccharide fractions was analyzed using High Performance Anion Exchange Chromatography with Pulsed Amperometric Detection (HPAEC-PAD) equipped with a gold electrode. The monosaccharides were eluted isocratically with 16 mM sodium hydroxide followed by the elution of the acid monosaccharides starting at 20 min with a linear gradient to 200 mM sodium hydroxide + 500 mM sodium acetate in 20 min. Data analysis was performed with Dionex Chromeleon software version 6.80. Quantitative analyses were carried out using standard solutions of the monosaccharides (rhamnose, galactosamine, glucosamine, galactose, glucose, mannose, xylose, galacturonic acid, and glucuronic acid) (Sigma-Aldrich, St. Louis, USA).

### Host cell signaling assay

Human embryonic kidney (HEK)-293 cells not expressing TLR receptors but harbouring pNIFTY, a NF-κB luciferase reporter construct (Invivogen, Toulouse, France) were used as the negative control in the NF-κB assays. HEK-293 cells (Invivogen, Toulouse, France) expressing human TLR2 and pNIFTY, a NF-κB luciferase reporter construct (Invivogen) were derived as previously described [50]. The HEK-293 TLR2 reporter cell line was seeded at 5 × 10^5^ cells/cm^2^ in 96-well plates and incubated overnight under standard culture conditions. Cells were then stimulated with 2 independently grown bacterial cultures of the *L plantarum* wild-type like derivative (NZ3400Cm) or *cps* deletion mutants (15 CFU/HEK-293 cell). After this incubation period, the medium was replaced with Bright-Glo™ (Promega Benelux BV, Leiden, The Netherlands), the plate was vortexed, and the luminescence was measured using a Spectramax M5 (Molecular Devices, Sunnyvale, USA). As positive control, the TLR2 agonist Pam3CysSK4 (5 μg/mL) was used, and as negative control, no bacterial cells were added to the HEK-293 cells.

## Competing interests

The authors declare that they have no competing interests.

## Authors’ contributions

DMR, RvK, PAB and MK drafted the manuscript. MK, JW and PAB supervised the project. DMR, IIvS, NT, and RSB performed the experiments, DMR and MK analyzed the data, RvK performed *cps* cluster analysis and comparison, MW designed the hybridization scheme of the microarray experiments and processed the transcriptome data. All authors read and approved the manuscript.

## Supplementary Material

Additional file 1 **Shared regulated genes between *cps *cluster mutant.** Click here for file

Additional file 2 **CPS biosynthesis-related genes of *L. plantarum *WCFS1 and their homologues in strains ATTC 14917, JDM1, and ST-III.** Click here for file

Additional file 3 Primers, plasmids, and strains used in this study. Click here for file
